# Trends in prevalence of hepatitis B virus infection among Albanian blood donors, 1999-2009

**DOI:** 10.1186/1743-422X-8-96

**Published:** 2011-03-04

**Authors:** Vjollca Durro, Shpetim Qyra

**Affiliations:** 1 Hospital Planning Directory, Ministry of Health, Bulevardi " Bajram Curri", No.1, Tirana, Albania; 2 Department of Chronic Diseases Epidemiology and policies, Public Health Institute Rruga" Aleksander Moisiu", No 30, Tirana, Albania

## Abstract

**Background:**

Hepatitis B virus (HBV) was among the first virus known to be transmitted by blood and blood productions. The objective of this study is to determine the trend of hepatitis B virus in blood donors.

**Materials and methods:**

In this study 79274 blood donors were retrospectively evaluated for HBsAg. The donors were selected using personal questionnaire, physical examination and testing blood before donation. Blood banks records are used as source of information. The blood donors samples were analyzed for the presence of hepatitis B surface antigen (HBsAg) by commercial available kits ELISA method, third generation (from Abbott laboratory, Germany). A sample was considered as HBsAg positive when found twice repeatedly reactive. Reactive samples were not confirmed with addition tests.

**Results:**

In the evaluation data, we found out that from 79274 of the total healthy blood donors, 15983 were voluntary donors, 52876 were family replacement donors and 10424 commercial blood donors. The prevalence of HBsAg in blood donors was 7.9%. It was increased steadily from 5.9% in 1999 to 9.1% in 2006 and decreased in 7.9% in 2009. According to blood donors status the HBsAg prevalence was 10.5% in commercial blood donors, 8.1% in voluntary donors and 8.6% in family replacement donors. The prevalence of anti-HBc in blood donors was 59.1%.

**Conclusion:**

The prevalence of HBsAg was lower in voluntary non remunerate blood donors than commercial donors and family replacement blood donors. In FDs the prevalence was higher than VDs but lower than CDs. So, it is important to encourage the voluntary blood donors to become regularly blood donors.

## Background

Hepatitis B virus (HBV) was among the first virus known to be transmitted by blood and blood products. HBV infection from transfusions became rare after the introduction of the HBsAg test in early 1970, but remains one of the most common serious complications of transfusion particularly in countries with high and intermediate prevalence of HBV [1-4]. The residual transmission risk of HBV through transfusion is higher [5-8]. This is attributed to the interval between initial HBV infection and the detection of hepatitis B surface antigen (HBsAg), resulting in a long window phase during which the virus is transmissible [9,10]. After the implementation of HBsAg screening in the 1975s, there have been no further measures in Albania to decrease the residual risk of HBV transmission, other than improving the sensitivity of the HBsAg assay. Development of sensitive assays to detect HBV-DNA showed that healthy HBsAg-negative donors who are anti-HBc positive, may harbour an occult HBV infection and maintain HBV-DNA sequences in their liver and blood, thus representing potential sources of HBV transmission [11,12]. Evaluation of data on the prevalence of HBV, among blood donors permits an assessment of the occurrence of infection in the blood donor population and consequently the safety of the collected donations. This study was performed to assess prevalence and trends of the viral hepatitis B during last decade (2000-2009 years), among blood donors in Tirana. Moreover, in a pilot study, we also explored the prevalence of anti-HBC among randomly selected HBsAg negative blood donor samples.

## Materials and methods

In this study 79274 blood donors were retrospectively evaluated for HBsAg. The donors were selected using personal questionnaire, physical examination and testing blood before donation (as Hb level, blood pressure, body weight, temperature). Blood banks records are used (personal donor sheet, donation's records) as source of information. A first time blood donors is a donors who has donated blood for first time and only once. A regular blood donors is donors who has donated blood more than two time during one year.

In this study family replacement donors and voluntary non remunerate blood donors are first time blood donors. We haven't regular blood donors from these groups. Family replacement donors are donors who have donated the same number of units of blood as of patient's requirements, despite the blood group. A friend or family member of the recipient donates blood to replace the stored blood used in a transfusion, ensuring a consistent supply.

To define the prevalence of HBV infection the number of positive donors for each years is divided by the total number of donors for each years.

### Laboratory testing

#### Tests for HBsAg

The blood donors samples were analyzed for the presence of hepatitis B surface antigen (HBsAg) by commercial available kits (as was presented in table 1). A sample was considered as HBsAg positive when found twice repeatedly reactive. Reactive samples were not confirmed with addition tests. All reactive donors were rejected from further blood donations.

**Table 1:** The HBsAg screening kits used 1999-2009.

Years	Screening kit	Manufacture	Sensitivity	Method
**1999**	Auszyme Monoclonal HBsAg	Quantum II ABBOT	Ad 04 ng/ml, ay 0.7 ng/ml	Manual

**2000**	Auszyme Monoclonal HBsAg	Quantum II ABBOT	Ad 04 ng/ml, ay 0.7 ng/ml	Manual

**2001**	Auszyme Monoclonal HBsAg	Quantum II ABBOT	Ad 04 ng/ml, ay 0.7 ng/ml	Manual

**2002**	Auszyme Monoclonal HBsAg	Quantum II ABBOT	Ad 04 ng/ml, ay 0.7 ng/ml	Manual

**2003**	IMx	ABBOT	Ad 0.22 ng/ml Ay 0.17 ng/ml	Semi automatic

**2004**	IMx	ABBOT	Ad 0.22 ng/ml Ay 0.17 ng/ml	Semi automatic

**2005**	IMx	ABBOT	Ad 0.22 ng/ml Ay 0.17 ng/ml	Semi automatic

**2006**	AxSYM HBsAg (MEIA)	ABBOT	Ad 0.15 ng/mL Ay 0.12 ng/mL ≤0.5 ng/mL	automated

**2007**	AXSYM CMIA	ABBOT	100%	automated

**2008**	Architect	ABBOT	100%	fully automate

**2009**	Architect	ABBOT	100%	fully automate

#### Tests for anti-HBc

The blood donor samples were analyzed for the presence of anti-HBc using third generation monoelisa kit anti HBC plus (Bio - Rad) with sensitivity 0.5 U PEI/ml for IgG dhe 8 U PEI/ml for IgM and specificity 99.91%

## Results

A total of 79274 blood donors have donated blood during 10 years. In the evaluation data, we found out that from the total 79274 healthy blood donors, 15983 were voluntary donors, 52876 were replacement donors and 10424 commercial blood donors. Table 2 shows the number of blood donors who have donated blood during 1999-2009 years. The number of blood donors was progressively increased during the years from 5233 in 1999 to 14352 in 2009. The number of commercial blood donors was decreased from 1714 in 1999 to 284 in 2009. The number of voluntary non remunerate blood donors was increased from 1200 in 1999 to 3639 in 2009 donors and family replacement blood donors from 2319 in 1999 to 10429 in 2009.

**Table 2:** Blood donors during 1999-2009

Blood donors	Years											
	**1999**	**2000**	**2001**	**2002**	**2003**	**2004**	**2005**	**2006**	**2007**	**2008**	**2009**	**Total number**

**Voluntary donors No**.	1200	1110	210	509	506	605	1369	2027	2330	2478	3639	15983
**%**	22.9	25.9	6	12.9	11.6	11.7	19.1	23.9	20.8	21.3	25.3	

**Family replacement donors No**.	2319	1810	1965	2224	2774	3464	4877	5840	8403	8771	10429	52876
**%**	44.3	42.2	56	56.7	63.5	67.1	68.4	69.2	74.9	75.4	72.6	

**Commercial donors No**.	1714	1365	1333	1199	1088	1091	891	584	493	382	284	10424
**%**	32.8	31.9	38	30.6	24.9	21.2	12.5	6.9	4.3	3.3	1.9	

**Total**	5233	4285	3508	3923	4368	5160	7137	8451	11226	11631	14352	79274

Table 3 shows the prevalence of HBsAg in blood donors between 1999-2009 years. The HBsAg was detected in 5933 blood donors out of 79274 blood donors screened. The prevalence of HBsAg in blood donors was 7.9%. It was increased steadily from 5.9% in 1999 to 9.1% in 2006 and decreased in 7.9% in 2009.

**Table 3:** HBsAg distribution in blood donors during 1999-2009

Years	Total number of blood donors tested	Positive blood donors	
		
		No	%
1999	5233	307	5.9

2000	4285	235	6.5

2001	3508	178	5.1

2002	3923	272	6.9

2003	4368	275	6.3

2004	5160	376	7.3

2005	7137	568	7.95

2006	8451	769	9.1

2007	11226	898	8

2008	11631	920	7.9

2009	14352	1135	7.9

Total	79274	5933	7.5

Table 4 shows the prevalence of HBsAg based on donor's status. HBsAg was detected in 60 first time commercial blood donors out of 561 firs time commercial blood donors tested (prevalence 10.5%); in 1298 first time voluntary non remunerate blood donors out of 15983 first time voluntary non remunerate blood donors voluntary tested (prevalence 8.1%); in 4543 family replacement blood donors out of 52876 family replacement blood donors tested (prevalence 8.6%).

**Table 4:** Distribution of HBsAg based on donor status.

Donor status	Screened	HBsAg	
		
	No	No	%
First time commercial donors	561	60	10.5

First time Voluntary blood donors	15983	1298	8.1

Family replacement blood donors	52876	4543	8,6

Table 5 shows the prevalence of anti-HBc in blood donors. Anti-HBc was detected in 133 blood donors out of 225 blood donors tested (prevalence 59.1%). According to blood donors groups anti-HBc was detected in 73 first time blood donors out of 103 blood donors tested (prevalence 70.8%); in 60 regular blood donors out of 122 blood donors tested (prevalence 49.1%).

**Table 5:** Anti-HBc prevalence in blood donors

Donor status	Screened	Anti-HBc	
		
	No	No	%
Regular blood donors	122	60	49.1

First time blood donors	103	73	70.8

Total	225	133	59.1

## Discussed

Viral Hepatitis, especially hepatitis B in Albania continues to be a public health problem because of Albania is the highly endemic area for hepatitis B. In Albania the prevalence of HBsAg is 19% and anti-HBc 62% [13]. The endemicity of hepatitis B is described by the prevalence of HBsAg in the general population of a defined geographical area, and it varies considerably globally: HBsAg prevalence's of >8% are typical of highly endemic areas [14-17]. One of the primary concerns of Health Department is the need to suppress the spread of this highly contagious. The national blood transfusion service in Albania is only responsible body for collection, processing, testing and distribution of blood and blood products under supervision of Ministry of Health. HBsAg screening of all blood donations was mandatory in national level, since 1975. In present study we analyzed the profile of blood donors and estimated the prevalence of HBsAg. Traditionally paid donors (commercial donors) were the main source of blood in Albania. The first efforts for the voluntary blood donation began in 1994 with a cooperation between National Blood Service and Red Cross. One of the most important problems of blood transfusion service in Albania has always been reliance on professional blood donors (paid blood donors) that still exist even if has been reduce significantly. According to the data used in this study, in 1999 commercial blood donors composed 32.8% of blood donors versus 1.9% in 2009. During last years blood donors profile has changed as result implementation of national strategy for blood safety, launched in 2005. Exclusion of paid donors was achieved by rejecting the paid donor for the first time. First time commercial blood donors were prohibited since 2008. This change resulted in a very high percentage of first time blood donors from voluntary blood donors and family replacement blood donors groups. During these years family replacement blood donors constitute the largest group of blood donors in Albania. Our data in this study showed that 72.6% of blood donors were family replacement blood donors(in 2009).

The first lines of defence protection to assure the safety of blood for transfusion are rejected from donation the candidate of blood donors who are undergoing acute infection. Careful history taking is very important because the vast majority of infectious virus transmitted by transfusion are those causing in apparent or sub clinical infections in blood donors [18,19]. The prevalence of HBsAg in blood donors population depends on some factor as the prevalence of this virus in general population, diagnose of hepatitis B infection and sensitivity of screening test.

**Figure 1:**
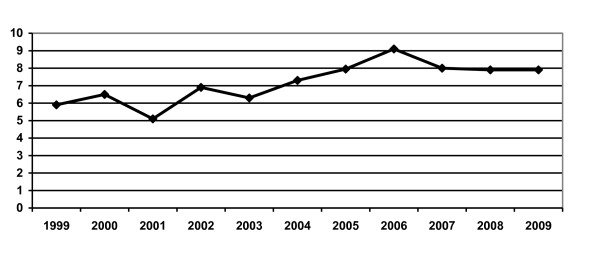
HBsAg prevalence in blood donors during 1999-2009 years

Our data (table 3/figure 1) showed the higher prevalence of HBsAg in blood donors, 7.9% (it ranging from 5.9% in 1999 to 7.9% in 2009) comparing with neighbor countries. So, the prevalence of HBsAg in blood donors population is 4,1% in Turkey, 0.4% in Greece, 4.2% in Kosovo, 1.32% in Macedonia, 0.4% in Italy [20-25].

The higher prevalence of HBsAg in blood donors reflects epidemiological situation of HBV in our country. According to previous studies in different group of Albanian population the prevalence of HBsAg were respectively in military recruitment 13,2%, in students 8-15.3% and pediatric aged 1.4 - 3.9% [26]. These data show that in blood donors the HBsAg prevalence is lower than general population because of pre-donation selection of blood donors who are interviewed with screening questionnaire.

As it is shown in the figure 1 there is an increase in prevalence of HBsAg in our donor population during years. This could be the result of the improved testing method from manual to fully automated and sensitivity of screening test(table 1) and with the constant increase of first time blood donors mainly family blood donors.

**Figure 2:**
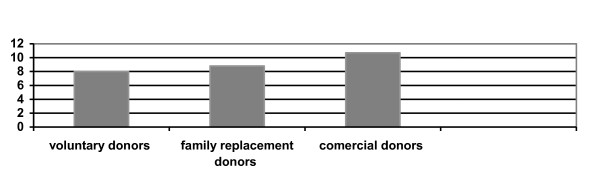
HBsAg prevalence based on blood donor status

According to donors status, the prevalence of HBsAg was lower in voluntary non remunerate blood donors than commercial donors and family replacement blood donors. In family replacement blood donors, the prevalence was higher than voluntary blood donors but lower than paid donors (figures 2). So, it is important to encourage the voluntary blood donors to become regularly blood donors. Screening the blood donors for anti-HBc or HBV DNA has reduced the risk of transmition of HVB infection by transfusion of blood and his products [27]. It is generally accepted that the diagnosis of infection by HBV is based on the presence of the HBsAg in the bloodstream [28]. However, screening of blood donors for HBsAg does not totally eliminate the risk of HBV infection through blood transfusion [29] since the absence of this marker in the serum does not exclude the presence of HBV DNA [30-33]. It is possible that, donors with occult HBV infection, who lacked detectable HBsAg but whose exposure to HBV infection was indicated by a positive anti-HBc and HBV DNA, are a potential source of HBV infection [34-36].

Our pilot study revealed that 133 of 225 (59.1%) HBsAg negative blood donors were anti-HBc positive and thus were exposed to HBV infection. The isolated finding of anti-HBc antibody in HBsAg-negative subjects was considered a marker of past exposure to HBV and of resolved infection. Results obtain from deferent studies in blood donors has shown that HBV DNA was detected in 1.6% to 38% of HBsAg negative/anti HBc positive blood donors [34,27,37]. Thus these donors my be the potential to transmit HBV through transfusion.

The absence of HBsAg in the blood of apparently healthy individuals may not be sufficient to ensure lack of circulating HBV. Blood containing anti-HBc with or without detectable presence of HBsAg might be infectious, therefore routine blood donor screening for anti-HBc has been implemented in some countries resulting in a decrease in the risk of post-transfusion HBV infection [38].

While anti-HBc-positive blood donor may be a potential source of HBV transmission, routine application of anti-HBc screening is not feasible in Albania. Our findings in random screening HBsAg negative blood donors for anti-HBc are the same with anti-HBc prevalence rates among HBsAg-negative blood donors in endemic area when the prevalence of anti-HBc is over 57% [39].

In country with intermittent and low prevalence of HBsAg, anti-HBc prevalence vary from, 0.56% in the United Kingdom, 0.84% in United States, 1.4% in Germany, 15.03% in Greece [10,34-38]. In our study the HBsAg negative/anti-HBc positive donor population is carry out in two blood donors groups, regular blood donors and first time blood donors. The prevalence rate of anti-HBc was higher in first time blood donors than regularly donors. So, the establishment of a panel of regular blood donors is very important in providing safe blood and blood products. Anti-HBc screening of blood donations is controversial and variably performed in different countries. Currently it is limited to areas where the seroprevalence of HBV is low (generally <2%), while it is not performed in areas with a high HBV seroprevalence because the impact of the deferral of anti-HBc-positive donors is considered not sustainable. However, the prevalence of occult HBV infection is higher in areas in which HBV infection itself is more frequent. The safety of this measure is currently being debated [40-42]. Our study underscores the increasing the HBsAg prevalence in our donor population. This trend, suggest that routine anti-HBc screening of blood donors could possibly prevent some transfusion-transmitted HBV infections from blood donors. However, usefulness of screening for anti-HBc in addition to HBsAg detection and introduction of PCR based screenings like NAT to improve the safety of the blood supply in Albania deserves further analysis.

## Competing interests

The authors declare that they have no competing interests.

## Authors' contributions

VD carried data collecting, participated in design of the study and drafted manuscript. SHQ participated in design of the study and performed the statistical analysis. Both authors read and approved the final manuscript.
